# Resolving conflict between aversive and appetitive learning of views: how ants shift to a new route during navigation

**DOI:** 10.3758/s13420-023-00595-z

**Published:** 2023-08-24

**Authors:** Vito A. G. Lionetti, Sudhakar Deeti, Trevor Murray, Ken Cheng

**Affiliations:** School of Natural Sciences, Macquarie University, Sydney, NSW 2109 Australia

**Keywords:** Ants Bull ants Navigation behavior Visual learning Context cues

## Abstract

Ants store and recall views associated with foraging success, facilitating future foraging journeys. Negative views are also learned, but instead prompt avoidance behaviors such as turning away. However, little is known about the aversive view’s role in navigation, the effect of cue conflict, or the contextual relationship between learning and recalling. In this study, we tested *Myrmecia midas’* capacity for aversive learning of views either independently of or in conflict with appetitive events. We either captured and released foragers when reaching a location or let them pass unhindered. After a few journeys, captured foragers exhibited aversive learning by circumventing the capture location and increasing both meandering and scanning. Ants that experienced foraging-appetitive and homing-aversive events on their journeys exhibited lower rates of avoidance behavior and scans than those experiencing aversive events in both outbound and homebound journeys. The foraging-aversive and homing-aversive ants exhibited similar levels of avoidance and scanning as those that experienced the foraging-aversive and homing-appetitive. We found that foragers showed evidence of context specificity in their scanning behavior, but not in other measures of aversive learning. The foragers did not increase their meandering and scans while approaching the views associated with aversive events. In addition to shedding light on the role of aversive views in navigation, our finding has important implications for understanding the learning mechanisms triggered by handling animals.

**Supplementary information:**

The online version contains supplementary material available at 10.3758/s13420-023-00595-z.

## Introduction

Although insects have a modest nervous system, they are able to perform a broad range of learned behaviors (Perry et al., 2013) from non-associative learning, such as habituation and sensitization (Braun & Bicker, 1992; Haupt & Klemt, 2005), to associative learning, such as classical conditioning (Menzel, 1993). Conditioning in insects has been shown in several learning protocols, such as in the proboscis extension response, where an unconditioned stimulus (sugar water proffered to the antennae) is paired with a conditioned stimulus (some odor), which leads to an extension of the proboscis (conditioned response) to the conditioned stimulus (Bitterman et al., 1983; Giurfa & Sandoz, 2012). In the foraging context, associative learning is crucial in recalling and locating foraging sites associated with positive rewards (Menzel, 1993). Insects can learn to associate a visual stimulus with a reward, a passage home (Schwarz & Cheng, 2010). Insects, however, can also associate negative stimuli with negative effects, a mechanism called *aversive learning* (Tully & Quinn, 1985). Honeybees were able to discriminate two different visual stimuli, where one stimulus was paired with an electric shock and the other stimulus with the absence of the shock (Mota et al., 2011). Desert ants learned views associated with negative experiences, enabling them to avoid those locations in the future (Wystrach et al., 2020). These foragers associated the negative experience of falling into and being delayed by a trap with the views experienced just before the event (Wystrach et al., 2020). To navigate safely and avoid negative experiences, foragers combine navigational cues and individual experience, shaped by both appetitive and aversive learning (Wystrach et al., 2020). While positive learning in insects is well established in the context of navigation, far less is known about aversive learning.

Learning plays a key role in ant navigation (Narendra et al., 2007) by providing environmental specificity and robustness (Collett, 1992; Philippides et al., 2011). Recent insect navigation models and experimental results suggest that during view learning, foragers learn not only attractive views, but also aversive/repellent views, which lead foragers to turn away from these views (Le Möel & Wystrach, 2020; Murray et al., 2020). According to these models, attractive and repellent views form two distinct memory banks. Current views are compared to attractive and repellent memory banks to obtain attractive and repellent familiarity values. The integration of these positive and negative values then modulates the magnitude of the foragers’ turns during their oscillatory path. Such models perform best and match experimental data most when both attractive and repellent views guide navigational behavior.

Aversive experiences lead to aversive memories that are independent from appetitive memories in terms of acquisition and storage (Klappenbach et al., 2022). Memories of aversive experiences vary in duration, even within the same species. For example, while honeybees that associated an odorant with an electric shock exhibited aversive responses for 72 h (Giurfa et al., 2009), they formed memories lasting less than 48 h when associating an odor with the bitter substance quinine (Klappenbach et al., 2022). The duration of aversive memories seems to depend on the strength of the aversive unconditioned stimulus and on other factors, such as the animals’ satiation level (Klappenbach et al., 2022). While appetitive learning and memory retrieval are known to be linked to context cues, it is unclear whether aversive learning is similarly affected by context.

Context cues, such as temporal and location cues, can have a large influence on decision making (Cheng, 2005; Collett et al., 1993; Gould, 1987; Rosas et al., 2013; Zhang et al., 2012). During navigation, Australian desert ants learn and use visual memories linked to their navigation-goal context (Wehner et al., 2006). The foragers in the study were not able to recognize visual cues learned in their habitual outbound route if these cues were experienced during their inbound route, suggesting that navigation cues are learned and recalled in association with their navigation-goal context. Thus far, all insect studies on the role of context concern appetitive learning, whereas it remains unclear how context cues influence aversive learning, and which neurological processes facilitate this aversive learning.

In the current study, we tested night-active bull ants *Myrmecia midas’* capacity for aversive learning in different navigational contexts. We set out first to establish that *M. midas* foragers exhibit aversive learning by exposing them to an aversive event at a specific location during both their inbound and their outbound trips. We predicted that aversive responses would manifest in avoiding the location of the aversive experience, and in increasing meander and scanning behaviors. We then tested whether the conflict between appetitive and aversive events reduced foragers' aversive responses and whether aversive learning is context specific. We used a capture-and-release procedure as an aversive event, and the absence of it as an appetitive event, when the foragers were either going off to forage or homing. We predicted aversive learning would be context-specific, causing aversive responses to be strongest when traveling in the direction in which the aversive event occurred. We also investigated whether the foragers increase their aversive behavior in advance of the area where they were captured. By looking at *M. midas*’ aversive learning, we aim to have a perspective on the impact of negative events on ants’ natural foraging behavior.

## Materials and methods

### Field site and study species

Experiments were conducted from September to December 2020 on *M. midas* nests located on the Macquarie University campus, Sydney, Australia. During evenings, *M. midas* foragers travel from the nest, located in the ground, to forage on nearby *Eucalyptus* trees, then return to the nest the following morning. *M. midas* foragers rely primarily on terrestrial landmarks when navigating in their environment to reach either their nest or a nearby foraging tree (Freas et al., 2017). Although in New South Wales no ethics approval is required to experiment on ants, we ensured that all the manipulations and tests on *M. midas* were non-invasive. The experimental procedure produced no long-term observable adverse effects, based on observations throughout the experiment and in future seasons. This nest and foragers have been used for multiple further studies since this experiment.

### Pre-experimental procedure

For the experiment, we selected a nest with sufficient foraging activity and that had two foraging trees at similar distances from the nest. To optimize foraging path recording, we removed vegetation on the ground along the foraging corridor and, using string and tent pegs, gridded the corridor with 1 × 1-m squares to improve recording accuracy. In the middle of the foraging corridor, we conceptually delimited an area of 250 × 20 cm, called the Trap area. As a precaution, we allowed all foragers to acclimatize to these changes for 2 days. To mark experimental ants, each forager was caught during its first appearance on the foraging tree and captured with a plastic vial. We then cooled the ants on ice for 5 min and individually marked them with a small amount of colored paint (Tamiya TM). The foragers were then released at their foraging tree, allowing them to continue foraging as normal. Throughout these experiments, we used red light to better observe foragers during their night-time foraging activities.

### Experimental procedure

**Fig. 1:**
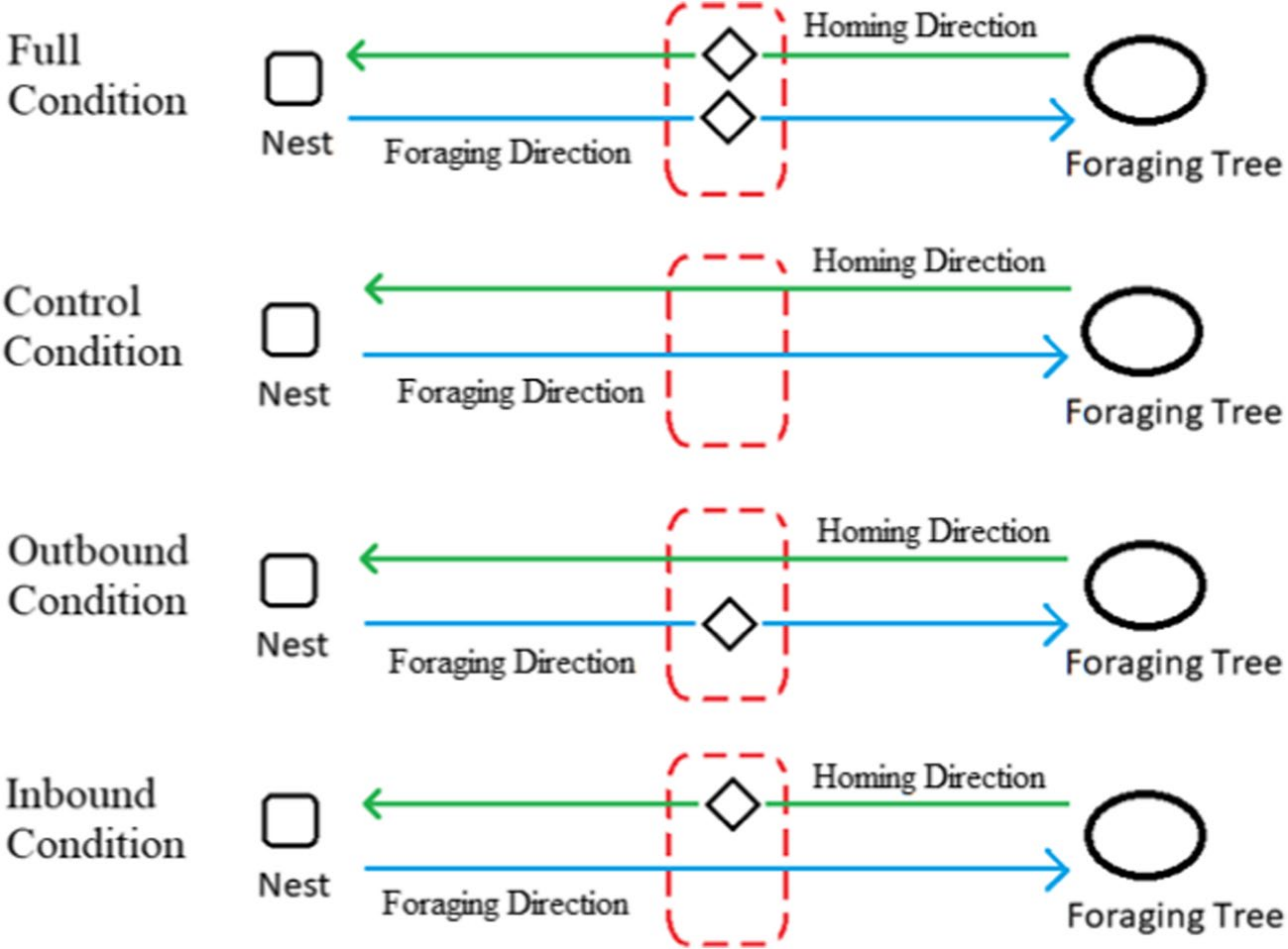
Illustration of the experimental procedures in the four conditions: Full Condition, Outbound Condition, Inbound Condition, and Control Condition. The blue and green lines represent the Foraging and Homing trips, respectively. The red dashed rectangular symbolizes the Trap area. The rhombus represents the capture-and-release procedure. Not to scale

The experiment consisted of four conditions: Full Condition, Outbound Condition, Inbound Condition, and Control (Fig. 1). Foragers were randomly assigned to one of these four conditions (n = 15 for each condition). All conditions followed the same basic procedure: foragers navigated from the nest to the foraging tree, called the Foraging direction; we caught, fed, kept them overnight, and then released them the following morning (7:00 – 9:00 a.m.); finally, they navigated from the foraging tree to the nest, called the Homing direction. It is worth noting that throughout this study, we did not specifically consider the foragers' response to the common event of being captured and restrained overnight. Each of the non-control treatments additionally involved the aversive event in which the forager was caught in the Trap area location using a darkened vial and kept for 10 s before being released and allowed to continue her journey from the same location. For the Outbound Condition, we performed this aversive capture only during the Foraging direction. In the Inbound Condition, we captured the forager only during their Homing trip. In the Full Condition, we performed the aversive capture during both their Foraging and Homing directions when they entered the Trap area.

Because learning is often enhanced during repeated exposures to the same stimulus, we tested each experimental forager for a total of four consecutive Foraging trips over multiple overnight foraging trips. Because foragers could emerge at overlapping times, two observers took turns recording the foragers’ path and scanning. When both observers were recording, the nest colony activity was temporarily blocked by shining white light (which these ants avoid) on the nest entrance until an observer was available.

### Data collection and processing

From the beginning of the experiment, we recorded every forager’s trip. We recorded the foragers’ paths and the location of scanning behaviors on graph paper, matched to our real-world grid. We converted the scans of these paths into multiple *x–y* coordinates using Web Plot Digitizer (https://automeris.io/WebPlotDigitizer/). From this digitized data, we analyzed the foragers’ paths and scanning behavior using R (R Core Team, 2021). We identified two categorical responses to the aversive treatments as avoidance behavior: Trap avoidance, and returning behavior, each of which diverges from normal foraging behavior in distinct ways. We measured Trap avoidance as foragers’ paths that did not cross into the Trap area but still reached the goal destination, either the nest location during Homing trips, or the foraging tree during Foraging trips. We measured the returning behavior as those forager’ paths that did not reach the goal destination and instead returned back to the starting point.

Beyond simple categorical measures of avoidance behavior, we also measured two secondary navigational behaviors that are known to vary in response to learning: scanning and path sinuosity. We counted the number of scans and calculated the meandering for each path. We defined a scan for each individual scanning bout, consisting of multiple saccadic body rotations and followed by a pause on the spot of at least 2 s. We calculated path meandering using the sinuosity of the foragers’ path, defined as a function of both the mean cosine of turning angles and step length (Benhamou, 2004). The function applies the formula “Sinuosity = 2[p(((1 + c)/(1 − c)) + b^2^)]^-0.5^”, where c is the mean cosine of turning angles, p and b are the expectation and the coefficient of variation of the step length, respectively. The sinuosity ranges between 0, a straight path, and 1, a highly curved path.

### Statistical analysis

For our statistical tests, we wanted to test the effect of our Treatment variables on our categorical and our secondary response variables. Our treatment variables are Condition (Control, Outbound, Inbound, and Full conditions), Trips (first, second, third, and fourth trips), Directions (Foraging and Homing directions), and Positions (Before and After Trap). We identified as Before and After Trap the aversive response that took place before and after encountering the Trap area, respectively. We used a binomial generalized linear mixed model to test the effect of Conditions, Trips, and Directions on our categorical variables. We used a linear mixed-model analysis of variance (ANOVA) to test the effect of Conditions, Trips, Directions, and Positions on our secondary response variables, scans and meandering. We used individual forager identification as a random factor in the binomial generalized linear mixed model and linear mixed-model analysis of variance. All models started by including each predictive variable and their three-way interactions. We then dropped non-significant terms and interactions sequentially based on the change in deviance and kept the model with the best Akaike Information Criterion (AIC). Once a final model was obtained, we made pairwise comparisons of the effect on each of our primary and secondary aversive responses using Tukey tests (alpha = 0.01). Since we tested multiple dependent variables, we adopted *p* = 0.01 as an alpha level to lower type 1 errors.

## Results

**Fig. 2:**
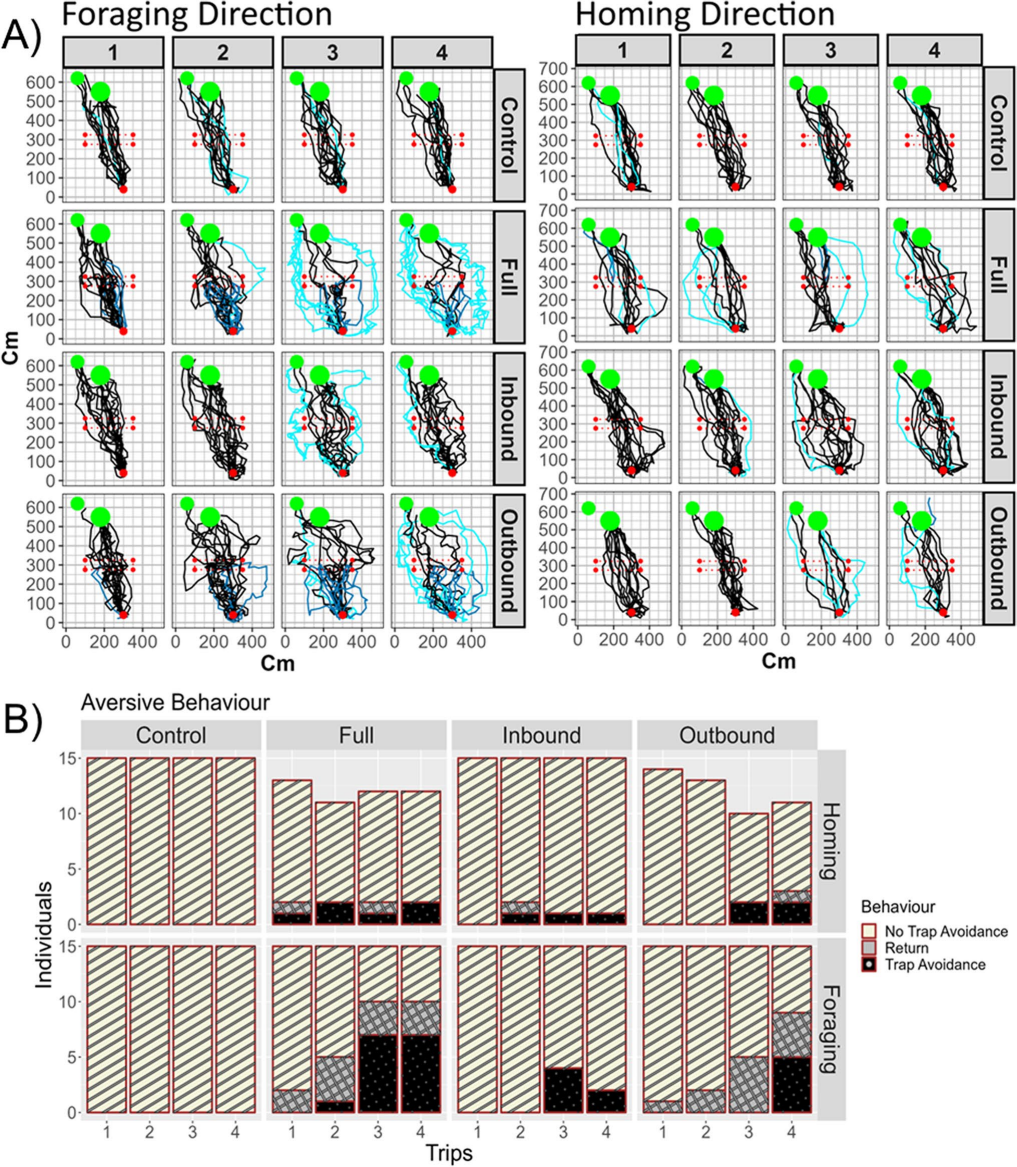
Individual Homing and Foraging paths of the foragers in the Control, Full, Inbound, and Outbound Conditions (**A**). We plotted one of every ten datapoints for each path. The black lines represent the foragers’ paths that did not avoid the Trap area. The light blue lines represent the foragers’ paths that avoided the Trap area. The blue lines represent the foragers’ paths that returned to the starting point. The red dot represents the nest. The red rectangle represents the Trap area. The green circles represent the foraging trees. Bar chart of the number of individuals performing aversive behaviors, Trap avoidance and Return, and no-aversive behavior, No trap avoidance, in the different conditions, trips, and directions (**B**)

**Fig. 3:**
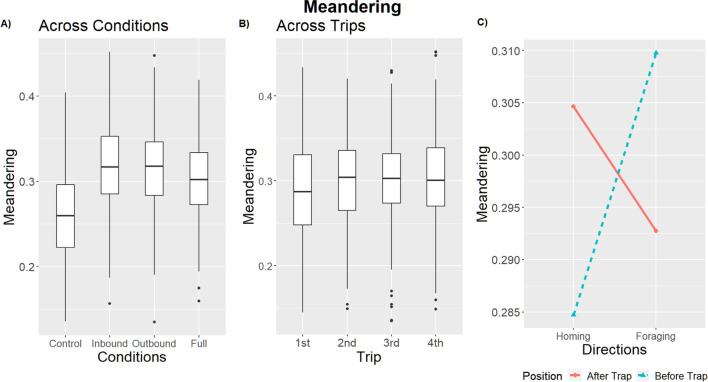
The box plots show the meandering (**A**) across conditions, and (**B**) across trips. The box plots mark the median with first and third quartiles and whiskers reach to 1.5× inter-quartile distance. Interactions in meandering with 95% confidence intervals between Position and Direction (**C**)

**Fig. 4:**
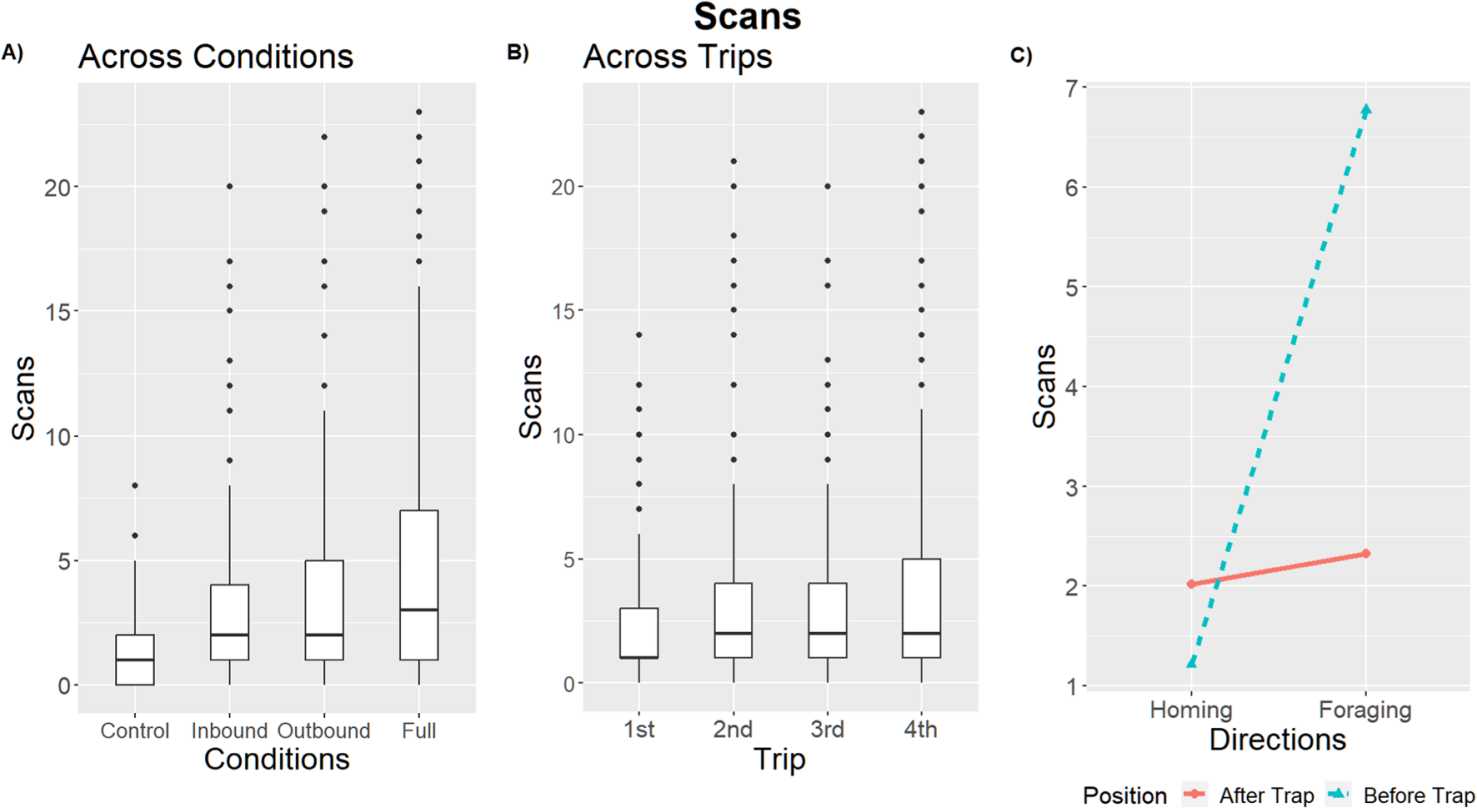
The box plots show the number of scans across conditions (**A**), and trips (**B**). The box plots show the median with first and third quartiles and whiskers reach to 1.5× inter-quartile distance. Interactions in scans with 95% confidence intervals between Position and Direction (**C**)

Undergoing aversive experiences caused foragers to increase aversive responses. We found significant increases in avoidance behavior (*p* < 0.01; Table 1, Fig. 2), meandering (Conditions: *p* < 0.01; Table 2, Fig. 3), and scans (Condition: *p* < 0.01; Table 3, Fig. 4) due to Condition, with all non-control treatments significantly increasing both scanning and meandering, and with Full and Outbound foragers increasing avoidance behaviors compared with Controls.

**Table 1:** Binomial generalized linear mixed models results for avoidance behavior (alpha = 0.01). Dropped variables are marked with and asterisk and we report the values immediately before being dropped

Avoidance behavior				
	Estimate	Std. Error	z value	*p* value
(Intercept)	6.079	0.878	6.924	**< 0.001**
Condition-Full	–3.666	0.781	–4.695	**< 0.001**
Condition-Inbound	–1.957	0.805	–2.432	0.015
Condition-Outbound	–2.989	0.785	–3.806	**< 0.001**
Direction-Foraging	–1.051	0.307	–3.426	**0.001**
Trip-2	–0.838	0.49	–1.709	0.087
Trip-3	–1.61	0.469	–3.433	**0.001**
Trip-4	–1.734	0.468	–3.705	**< 0.001**
* Condition-Full: Direction-Foraging	–1.243	1.868	–0.665	0.506
* Condition-Inbound: Direction-Foraging	0.070	1.955	0.036	0.971
* Condition-Outbound: Direction-Foraging	–0.814	1.898	–0.429	0.668
* Trip-2: Direction-Foraging	–0.562	1.084	–0.519	0.604
* Trip-3: Direction-Foraging	–2.157	1.040	–2.075	0.038
* Trip-4: Direction-Foraging	–1.973	1.025	–1.924	0.054

**Table 2:** Linear mixed models result for meandering (alpha = 0.01). Dropped variables are marked with an asterisk, and we reported the values immediately before being dropped

Meandering						
term	sumsq	meansq	NumDF	DenDF	F value	p value
Condition	0.051	0.017	3	56.205	10.968	**< 0.001**
Position	< 0.001	< 0.001	1	819.01	0.301	0.584
Trip	0.038	0.013	3	821.813	8.062	**< 0.001**
Direction	0.01	0.01	1	819.021	6.121	0.014
Position:Direction	0.075	0.075	1	819.045	48.264	**< 0.001**
Trip:Direction	0.014	0.005	3	818.796	3.005	0.03
* Condition:Direction	0.005	0.002	3	807.146	0.975	0.404

**Table 3:** Linear mixed models result for scans (alpha = 0.01)

Scans						
term	sumsq	meansq	NumDF	DenDF	F value	p value
Condition	649.239	216.413	3	59.975	29.706	**< 0.001**
Position	740.330	740.330	1	780.510	101.622	**< 0.001**
Trip	445.465	148.488	3	788.874	20.382	**< 0.001**
Direction	1897.992	1897.992	1	792.420	260.530	**< 0.001**
Condition:Position	205.351	68.450	3	780.510	9.396	**< 0.001**
Condition:Trip	198.129	22.014	9	788.568	3.022	**0.001**
Condition:Direction	556.321	185.440	3	792.047	25.455	**< 0.001**
Position:Trip	122.034	40.678	3	780.510	5.584	**0.001**
Position:Direction	1528.212	1528.212	1	780.510	209.772	**< 0.001**
Trip:Direction	331.510	110.503	3	788.814	15.168	**< 0.001**
Condition:Position:Trip	33.857	3.762	9	780.510	0.516	0.863
Condition:Position:Direction	381.721	127.240	3	780.510	17.466	**< 0.001**
Condition:Trip:Direction	118.700	13.189	9	788.512	1.810	0.063
Position:Trip:Direction	90.499	30.166	3	780.510	4.141	**0.006**
Condition:Position:Trip:Direction	53.394	5.933	9	780.510	0.814	0.603

### Aversive versus appetitive and aversive

While our Full-condition foragers were exposed to the most aversive stimuli in our experiment, the extent of their aversive response was not always higher than those of foragers in the intermediate conditions: Inbound and Outbound. Full-condition foragers had the highest rate of avoidance behavior (Trap avoidance and returning behaviors), although it was not significantly higher than Outbound foragers (Avoidance: 13 of 15; post hoc comparison, Control – Full condition: *z* ratio = –4.69, *p* < 0.01; Full – Outbound: *z* ratio = 1.79, *p* = 0.28; Full – Inbound: *z* ratio = 3.96, *p* < 0.01; Table S1 and Fig. S2 in the Online Supplementary Material (OSM)), and their meandering was not higher than either Inbound- or Outbound-condition foragers (post hoc comparison, Control – Full: *t* = –3.51, *p* < 0.01; Full – Inbound: *t* = –1.40, *p* = 0.50; Full – Outbound: *t* = –1.44, *p* = 0.48, Table S2 (OSM), Fig. 3a). Full-condition foragers showed more scans than Inbound and Control foragers, with only a trend compared with Outbound foragers (post hoc comparison, Control – Full condition: *t* = –9.18, *p* < 0.01; Full – Outbound: *t* = 2.81, *p* = 0.04; Full – Inbound: *t* = 4.20, *p* < 0.01; Table S3 (OSM), Fig. 4a).

Outbound-condition forgers also showed significant increases in all measures of aversion relative to controls (Avoidance: 12 of 15; post hoc comparison, Control – Outbound: z = –3.81, *p* < 0.01; Table S1 (OSM); Meandering: post hoc comparison, Control – Outbound: *t* = –4.97, *p* < 0.01; Table S2 (OSM); Scanning: post hoc comparison, Control – Outbound: *t* = –6.32, *p* < 0.01; Table S3 (OSM)), but were never significantly different from those of Inbound foragers (Avoidance: post hoc comparison, Inbound – Outbound: z = –2.33, *p* = 0.09; Table S1 (OSM); Meandering: post hoc comparison, Inbound – Outbound: *t* = –0.05, *p* = 1; Table S2 (OSM); Scanning: post hoc comparison, Inbound – Outbound: *t* = –1.33, *p* = 0.55; Table S3 (OSM)). Inbound foragers showed significantly higher rates of meandering and scanning than the Control foragers (Meandering: post hoc comparison, Control – Inbound: *t* = –4.95, *p* < 0.01; Table S2 (OSM); Scanning: post hoc comparison, Control – Inbound: *t* = –5.20, *p* < 0.01; Table S3 (OSM)), but did not differ significantly from Controls in avoidance behavior (Avoidance: 7 of 15; post hoc comparison, Control – Inbound: *z* = –2.43, *p* = 0.07; Fig. S1 (OSM), Table S1 (OSM)). Although Control ants never exhibited avoidance behavior (0 of 15; Fig. S1 (OSM)), they did exhibit baseline rates of scanning and meandering (Average scans per trip = 2.44; Average meandering per trip = 0.26; Fig. 3, Fig. 4).

### Context: Capture and recall

We explored the effect of capturing foragers only when traveling Outbound or only when traveling Inbound over multiple trips, on the extent of their aversive response when traveling in the same or opposite direction on subsequent trips. We found that foragers exhibited higher rates of aversive behaviors in the Foraging direction (Direction: aversive response: *z* = -3.43, *p* < 0.01; meandering: *z* = 6.12, *p* < 0.05; scans: *z* = 260.53, *p* < 0.01; Tables 1, 2, and 3). We found that foragers exhibited significantly higher avoidance behavior in the foraging direction regardless of the condition they underwent (Condition*Direction: *p* > 0.5, Table 1). In addition, experiencing different conditions did not make the foragers exhibit significant different meandering in their Foraging and Homing trips (Condition*Direction: *F* = 0.97, *p* = 0.4; Table 2).

Despite the lack of an effect of context on avoidance and meandering, we did find such an effect on Scanning (Condition*Direction: *F* = 25.5; *p* < 0.01; Table 3; Fig. S2a (OSM)). While the amount of scanning of both Inbound and Outbound ants increased when traveling in the Foraging direction, this increase was far less for Inbound than Outbound ants (post hoc comparison scans Homing – Foraging direction: Inbound condition, *t* = –6.42; *p* < 0.01; Outbound condition, *t* = –11.15; *p* < 0.01; Table S4 (OSM)) leading to lower Foraging-direction scans for Inbound ants (post hoc comparison, Foraging direction with contrast Inbound – Outbound conditions, *t* = –3.37; *p* < 0.01; Table S5 (OSM)) and no significant difference in scans when homing (Homing direction with contrast Inbound – Outbound conditions, *t* = 1.11; *p* = 0.68; Table S5 (OSM)). By comparison, in all non-control treatments, foragers scanned significantly more often in the Foraging direction than in the Homing direction (post hoc comparison scans Homing – Foraging direction: Full condition, *t* = –12.5; *p* < 0.01; Inbound condition, *t* = –6.42; *p* < 0.01; Outbound condition, *t* = –11,15; *p* < 0.01; Fig. S2a and Table S4 (OSM)); whereas control ants showed no detectable increase in scanning (post hoc comparison scans Homing – Foraging direction: Control condition, *t* = –1.85, *p* = 0.06, Fig. S2a and Table S4 (OSM)).

### Learning to predict or reacting?

While avoidance obviously requires anticipation of the trap and scanning may facilitate such avoidance, we found evidence that meandering and scanning increase before encountering the Trap area in the Foraging direction, but not in the Homing direction (meandering Interaction effect: Position*Direction, *F* = 48.26, *p* < 0.01; Table 2, Fig. 3; scans Position*Direction, *F* = 209.77, *p* < 0.01; Table 3, Fig. 4, and Fig. S3 (OSM)). The foragers performed more meandering (post hoc comparison After – Before Trap: Homing direction, *t* = 5.23*, p* < 0.01; Foraging direction, *t* = -4.58, *p* < 0.01; Table S6 (OSM)) and scanning (post hoc comparison After – Before Trap: Homing direction, *t =* 3.02, *p* = 0.01; Foraging direction, *t =* -17.97, *p* < 0.01; Table S7 (OSM)) in the Before Trap area in the Foraging direction, but in the After Trap area when traveling in the Homing direction. Position alone had no significant effect on meandering (position: *F* = 0.3, *p* = 0.584; Table 2), and while it does have a significant effect on scanning, the extent of this effect is overshadowed by its interaction with Direction (Position: *F* = 101.62, *p* < 0.01; Table 3, Fig. 4).

### Aversive response over trips

We also collected data on the effect of the repeated aversive experiences that intensified the magnitude of the foragers’ aversive reactions (Figs. 2, 3, and 4). Over multiple trips foragers showed a significant increase in aversive responses (Table 1), meandering (Trip: *F* = 8.06, *p* < 0.01; Table 2), and scans (Trip: *F* = 20.38, *p* < 0.01; Table 3). In addition, changes to the foragers' scanning behavior over trips was dependent on the traveling direction (Fig. S2b (OSM)): While the foragers’ scanning behavior increased over trips in the Foraging direction, it stayed stable over trips in the Homing direction (Interaction effect: Trip*Direction, *F* = 15.17, *p* < 0.01; Table 3 and Table S8 (OSM)). In addition, a similar interaction was detected between Trip and Position, which shows that successive exposures to aversive experiences increase the number of scans more in the Before Trap area than in the After Trap area regardless of whether capture-and-release was involved in the Foraging, Homing, or both directions or no-physical-manipulations were involved in the foragers’ trips (Interaction effect: Position*Trip, *F* = 5.58, *p* < 0.01; Fig. S2c (OSM), Table 3, and Table S9 (OSM)).

## Discussion

We investigated how foraging ants respond to being captured and then released at the same location over successive trips. We found evidence of aversive learning in the form of foragers avoiding the capture-and-release location, and increasing rates of scanning and meandering. In the proximity of the location associated with a negative experience, foragers had a tendency to take an indirect path that avoided the Trap area (Fig. 2). When the location was associated with a positive foraging and a negative homing experience, the foragers exhibited lower rates of avoidance behavior and scans; however, that was not the case when the direction of travel associated with the positive and negative experiences was reversed. We expected to have an increase in aversive responses in the direction in which the foragers experienced the aversive event, but found instead an increase in these behaviors in the Foraging direction. In addition, the foragers showed an increase in aversive response on the nest-half side of the foraging corridor, regardless of the travel direction. The foragers showed more scans and meandering after the Trap in the Homing direction, and before the Trap in the Foraging direction.

### Sensitivity towards aversive events

The *M. midas* foragers in this study treated the capture-and-release procedure as an aversive experience. In contrast, *M. bagoti* do not show any avoidance behavior or any difference in navigation performances after capture-and-release events (Freas & Cheng, 2018; Wystrach et al., 2019) or even after more invasive procedures, such as partial amputation of legs (Wittlinger et al., 2006). So why do *M. midas* foragers react so strongly to this minimally invasive capture-and-release procedure, when desert ants do not? The explanation could lie in *M. midas*’ longevity. *Myrmecia pyriformis* foragers have been observed to live over 3 years (Dietemann et al., 2004), as compared to an average life span of five foraging days after emergence from the nest in desert ants (Muser et al., 2005). Due to this longer life span, each forager of *M. midas* is more worth preserving compared with desert ants. *M. midas’* greater sensitivity to external events may be a strategy to protect the colony’s investment in the foraging force. Such sensitivity comes, however, at a cost in the form of increased sensory and cognitive capacity to detect and learn from such events (Mery, 2013). The generality and validity of this longevity–sensitivity link require further studies comparing cognition and longevity across diverse species in ants and across Animalia more broadly.

### Conflict between positive and aversive events

In the Inbound condition, with positive foraging experience but aversive homing experiences, *M. midas* foragers showed a lower level of trap avoidance and scans compared with experiencing aversive events in both directions (Full condition), but aversive experiences in the Foraging but not the Homing direction (Outbound condition) produced similar levels of trap avoidance and scans as the Full condition (Figs. 2 and 4). We assume that foragers had experienced over the experiment an interplay of appetitive and aversive learning of the views associated with a specific location. It has been shown in vertebrate animals that the association of an aversive unconditioned stimulus with a neutral stimulus evokes a fear response when later exposed to the, now, conditioned response (Fear conditioning) (Sanders et al., 2003). However, the introduction of the conditioned stimulus without the previous aversive unconditioned stimulus will produce a response reduction, called extinction (Vurbic & Bouton, 2011). We cannot explain this result based solely on competition between appetitive and aversive learning of specific stimuli since the foragers in the Outbound and Inbound conditions both experienced half as many aversive experiences as those in the Full condition, but behave differently. One possibility is that the difference lies in performance rather than learning. The aversive experiences on outbound in inbound journeys were equally aversive, but other factors drove different behaviors (see subsection *Responding in different predation pressure*).

### Learning and responding in a specific context

Across multiple measures of scanning behavior, meandering, and aversive responses, the foragers showed only one case of context specificity in aversive learning. The Inbound foragers had a lower increase in scanning in the Foraging direction than Outbound foragers (Fig. S3a (OSM)). It must be remarked, however, that the Inbound and Outbound conditions did not differ significantly in scanning in the Homing directions by a post hoc test, throwing doubts on any claim of context specificity in learning. In *M. bagoti*, foragers did not generalize the views experienced in the Foraging-direction context to the Homing-direction context, although context specificity in the Foraging direction was not tested (Wehner et al., 2006). Context specificity in *M. bagoti* was also found when ants were repeatedly captured and returned to an earlier part of their route, a procedure called *rewinding* (Wystrach et al. 2019). Rewinding disturbed navigational performance, resulting in more scanning and meandering, especially during the section in which the ants were rewound. Differences in species and procedures preclude any firm conclusions, so that further testing of context specificity in aversive learning needs to be done.

### Responding in distinct locations

*M. bagoti* foragers showed a similar increase in meandering and scans in the region before encountering the aversive location, although they were only tested in the homing context (Wystrach et al., 2020). After the trap, they traveled in a “business-as-usual” fashion. Homing *M. midas* foragers, in contrast, showed an increase in scans and meandering after crossing the trap area (Figs. 3 and 4), although this effect was larger in meandering than in scans. Foraging *M. midas* foragers, on the other hand, showed an increase in scans and meandering before encountering the trap area (Figs. 3 and 4), the before-aversive-experience characteristic resembling the effect found in *M. bagoti*. One proposed mechanism is that the foragers change the valence of the view memories experienced just before the aversive experience (Wystrach et al., 2020), perhaps by labeling specific cues preceding an aversive event (Bouton, 2007). Such an account explains the behavior found in *M. midas* traveling in the Foraging direction but not in the Homing direction. The unpredicted behavior of *M. midas* in the Homing direction may be due to a combination of the challenge of finding the nest and perceived risk, discussed next.

### Responding in different predation pressure

In the Homing direction, the foragers showed a lower response to the aversive learning, taking a more direct route, disregarding the Trap area more, and performing fewer scans. In contrast, in the Foraging direction, foragers showed a greater number of alternative route-taking and scanning behaviors. We speculate that the difference in the number of scans between the Homing and Foraging directions is due to the brighter light conditions present on tests in the Homing direction increasing the perceived risk of predation relative to the risk of trapping. On the other hand, low-light conditions produce a decrease in foragers’ visual perception (Narendra et al., 2013), possibly requiring more scanning. Brighter light raises the risk of predation from non-experimental sources such as birds and lizards. While still perceiving the trap as aversive (learning), the animals might risk the trap more in order to get home quicker (a performance factor), along with increased scanning for predators. Meandering from side to side usually goes with scanning because the lateral turns also lead to a wider surveillance of the route of travel.

### Aversive reinforcement

Repeated aversive experiences induce a stronger foragers’ response in trap avoidance, meandering, and scanning. Latent inhibition, where previous unconditioned experiences inhibit subsequent learning, may explain why multiple aversive experiences were needed for foragers to learn to avoid the Trap area (Bouton, 2007; Chandra et al., 2000). In the current study, a view associated with numerous successful journeys might require multiple negative experiences to change valence. Once the negative experience was avoided, the forager’s new path was reinforced by a positive association. This is consistent with a previous study on aversive learning in desert ants where the foragers were free to visit the feeder but experienced a pit trap on their homebound journey (Wystrach et al., 2020). In that study, 28% of the homing foragers were able to avoid the trap after 24 h of training, compared to this study, where 40% of the non-control treatment foragers showed at least one aversive response in the Homing direction in the Full condition (Fig. S1 (OSM)). However, only in 13% of the foragers’ inbound trips did the ants show an aversive response. We caution against making any conclusions about species differences in aversive learning propensities, however, due to the variation in experimental procedures.

### Neural aspect and implications

The results of this study showed that foragers’ behavior is not simply a response to present conditions but is dependent on their experiences and learning. In insects, the mushroom bodies, cerebellum-like structures counterpart in vertebrates (Farris, 2011), support learning and memory formation (Zars, 2000). Visual information is transported from the optic lobes to the mushroom bodies via projection neurons (Habenstein et al., 2020). This information is stored in the mushroom bodies, represented as a specific pattern of activated Kenyon cells (Ardin et al., 2016). In recall, the visual information stored in Kenyon cells is conveyed to the mushroom bodies’ output neurons (Aso et al., 2014). Learning modulates the synapses between the Kenyon cells and mushroom bodies’ output neurons (Webb & Wystrach, 2016). In this neural framework, the individual positive or aversive learning experience plays a role by increasing or decreasing the strength of the connection from the Kenyon cells to the mushroom bodies’ output neurons. We assume that individual learning plays a role by changing the weight given to different elements in the insect nervous system. Repeated capture-and-release procedures produced aversive learning towards the view paired with the negative experience, leading the foragers to avoid the repellent view, as in the Full Condition. The multiple experiences required by the foragers to avoid the Trap area suggest a gradual operation of the dopaminergic neurons. The dopaminergic neurons modulate the valence of the views experienced by regulating the synapses between the Kenyon cells and mushroom body output neurons. Instead, pairing the view in alternation with negative and successful experiences produced a weakened response, although not significatively different in Outbound foragers. This result suggests both appetitive and aversive learning are taking place in an antagonist fashion.

## Conclusion

We showed that *M. midas* foragers were able to learn to avoid views associated with a negative outcome. Such a mechanism allows foragers to avoid future negative outcomes by taking alternative routes. The different response in the short-living desert ants to a similar manipulation suggests a potential ant longevity–sensitivity correlation, a suggestion that requires further testing. Furthermore, the foragers showed little evidence that aversive learning is dependent on the navigation-goal context of the aversive event, partially generalizing the aversive response to new contexts. The foragers also showed an increase in aversive response in the nest-half side of the foraging corridor, independently of whether it was before the Trap or after the Trap. We suggested different explanations for behaviors in different directions. Here we showed how even a mild physical handling can trigger aversive learning causing a complex pattern of responding.

### Supplementary information

ESM 1 (DOCX 4036 kb)

## Data Availability

https://mqoutlook-my.sharepoint.com/:u:/g/personal/vito_lionetti_hdr_mq_edu_au/Eatz4moaJmFItdvGrrzkJs8B6OQIiXgBIKXr1H6NU5Akwg?e=h462Iq
