# Supplementary material for: Resolving conflict between aversive and appetitive learning of views: how ants shift to a new route during navigation
Source: Learn Behav. 2023 Aug 24;51(4):446–57. doi: 10.3758/s13420-023-00595-z (PMC10716056; doi:10.3758/s13420-023-00595-z)
Supplement: Supplementary file 1 — (DOCX 4036 kb) [file 13420_2023_595_MOESM1_ESM.docx]

(R Core Team , 2021)**Supplementary Materials**

**Statistical analysis**

We transformed the value of a random individual in each condition to overcome the absence of variance in trap avoidance and returning behavior in the Control condition.


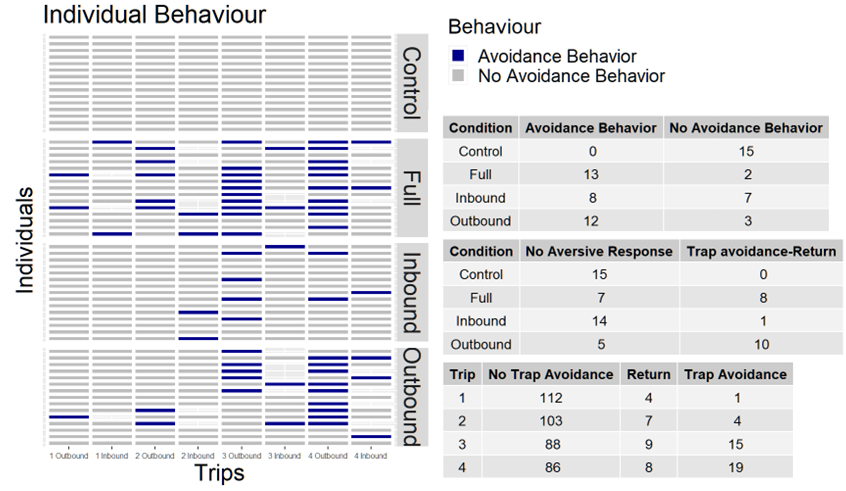


**Figure S1:** Tabulation of individuals’ behavioral responses over trips (A). Each row represents a specific forager. Table of the number of individuals performing no aversive responses or performing at least one aversive response (B).


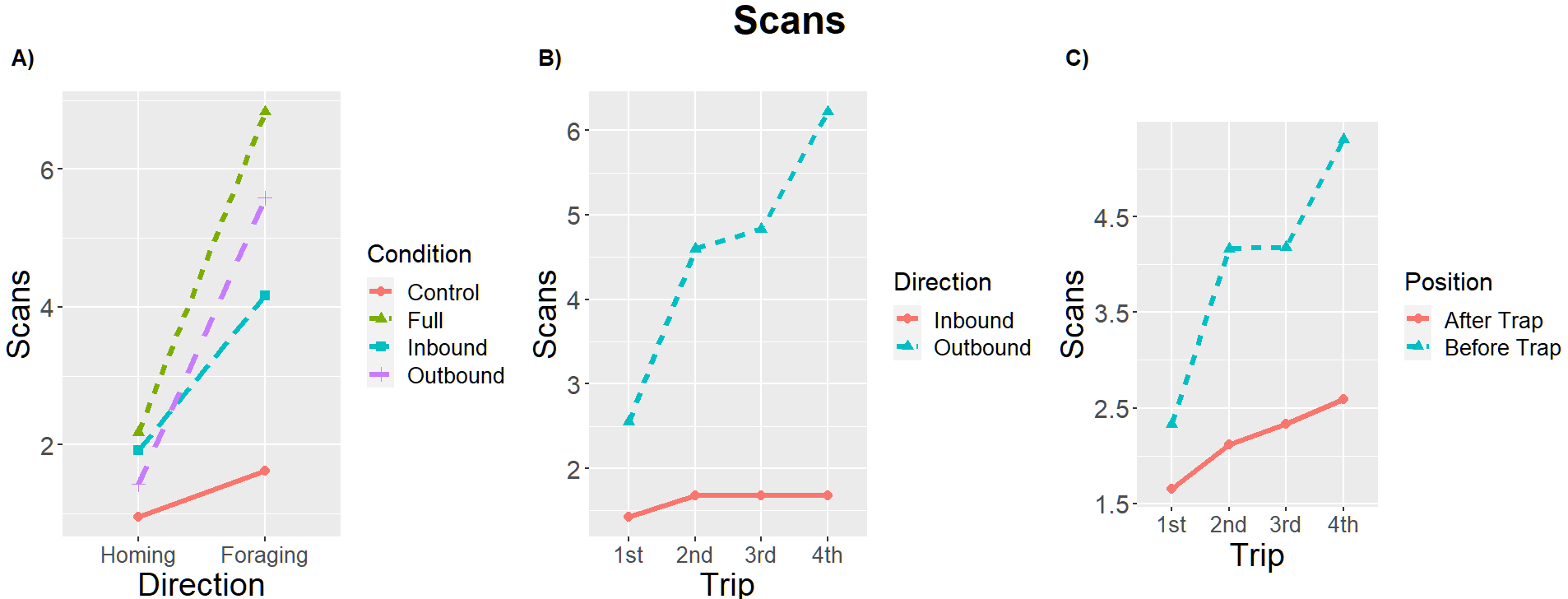


**Figure S2:** Interaction in Scans with 95% confidence intervals between Condition and Direction (A), Direction and Trip (B), and Position and Trip (C).


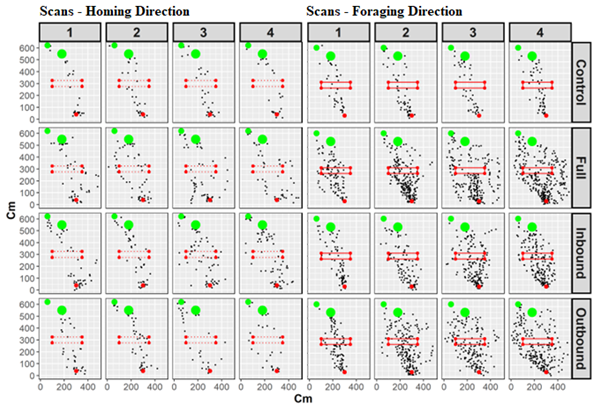


**Figure S3:** Scan positions of the foragers in the Control, Full, Inbound, and Outbound Conditions during the Homing and Foraging directions. The red dot represents the nest. The red rectangle represents the Trap area. The green circles represent the foraging trees.

**Table S1:** Post hoc comparisons of statistical results for avoidance behavior across conditions (alpha = 0.01).

| **Avoidance Behavior** | | | | | |
| --- | --- | --- | --- | --- | --- |
| **contrast** | **estimate** | **SE** | **df** | **z ratio** | **p value** |
| Control – Full | 3.666 | 0.781 | Inf | –4.695 | **< 0.001** |
| Control – Inbound | 1.957 | 0.805 | Inf | –2.432 | 0.071 |
| Control – Outbound | 2.989 | 0.785 | Inf | –3.806 | **< 0.001** |
| Full – Inbound | –1.709 | 0.432 | Inf | 3.957 | **< 0.001** |
| Full – Outbound | –0.678 | 0.378 | Inf | 1.794 | 0.276 |
| Inbound – Outbound | 1.032 | 0.442 | Inf | –2.334 | 0.090 |

Table S2: Post hoc comparisons of statistical results for scans across conditions (alpha = 0.01).

| **Meandering** | | | | | |
| --- | --- | --- | --- | --- | --- |
| **contrast** | **estimate** | **SE** | **df** | **t ratio** | **p value** |
| Control – Full | –0.040 | 0.011 | 56.038 | –3.514 | **0.005** |
| Control – Inbound | –0.056 | 0.011 | 54.778 | –4.946 | **< 0.001** |
| Control – Outbound | –0.056 | 0.011 | 55.967 | –4.968 | **< 0.001** |
| Full – Inbound | –0.016 | 0.011 | 56.060 | –1.403 | 0.503 |
| Full – Outbound | –0.016 | 0.011 | 57.210 | –1.444 | 0.477 |
| Inbound – Outbound | –0.001 | 0.011 | 55.993 | –0.049 | 1.000 |

**Table S3:** Post hoc comparisons of statistical results for meandering across conditions (alpha = 0.01).

| **Scans** | | | | | |
| --- | --- | --- | --- | --- | --- |
| **contrast** | **estimate** | **SE** | **df** | **t ratio** | **p value** |
| Control – Full | –3.221 | 0.351 | 59.372 | –9.179 | **< 0.001** |
| Control – Inbound | –1.754 | 0.337 | 69.366 | –5.201 | **< 0.001** |
| Control – Outbound | –2.218 | 0.351 | 59.693 | –6.320 | **< 0.001** |
| Full – Inbound | 1.466 | 0.349 | 58.226 | 4.200 | **< 0.001** |
| Full – Outbound | 1.002 | 0.356 | 58.810 | 2.813 | 0.040 |
| Inbound – Outbound | –0.464 | 0.349 | 58.542 | –1.329 | 0.548 |

**Table S4:** Post hoc comparisons of statistical results for scans across directions in the different conditions (alpha = 0.01).

| **Scans** | | | | | | |
| --- | --- | --- | --- | --- | --- | --- |
| **Condition** | **contrast** | **estimate** | **SE** | **df** | **t ratio** | **p value** |
| Control | Homing – Foraging | –0.663 | 0.357 | 789.474 | –1.855 | 0.064 |
| Full | Homing – Foraging | –4.656 | 0.372 | 802.621 | –12.503 | **< 0.001** |
| Inbound | Homing – Foraging | –2.246 | 0.350 | 782.134 | –6.416 | **< 0.001** |
| Outbound | Homing – Foraging | –4.160 | 0.373 | 794.792 | –11.154 | **< 0.001** |

**Table S5:** Post hoc comparisons of statistical results for scans across conditions in the different directions (alpha = 0.01).

| **Scans** | | | | | | |
| --- | --- | --- | --- | --- | --- | --- |
| **Direction** | **contrast** | **estimate** | **SE** | **df** | **t ratio** | **p value** |
| Homing | Control – Full | –1.224 | 0.446 | 146.132 | –2.743 | 0.034 |
| Homing | Control – Inbound | –0.962 | 0.421 | 162.925 | –2.284 | 0.106 |
| Homing | Control – Outbound | –0.470 | 0.447 | 148.646 | –1.052 | 0.719 |
| Homing | Full – Inbound | 0.262 | 0.443 | 143.142 | 0.590 | 0.935 |
| Homing | Full – Outbound | 0.754 | 0.462 | 154.632 | 1.631 | 0.364 |
| Homing | Inbound – Outbound | 0.493 | 0.444 | 145.624 | 1.110 | 0.684 |
| Foraging | Control – Full | –5.217 | 0.425 | 126.139 | –12.287 | **< 0.001** |
| Foraging | Control – Inbound | –2.546 | 0.418 | 158.912 | –6.085 | **< 0.001** |
| Foraging | Control – Outbound | –3.967 | 0.425 | 126.139 | –9.343 | **< 0.001** |
| Foraging | Full – Inbound | 2.671 | 0.422 | 123.226 | 6.331 | **< 0.001** |
| Foraging | Full – Outbound | 1.250 | 0.423 | 116.778 | 2.956 | 0.019 |
| Foraging | Inbound – Outbound | –1.421 | 0.422 | 123.226 | –3.369 | **0.005** |

**Table S6:** Post hoc comparisons of statistical results for meandering across positions in different directions (alpha = 0.01).

| **Meandering** | | | | | | |
| --- | --- | --- | --- | --- | --- | --- |
| **Direction** | **contrast** | **estimate** | **SE** | **df** | **t ratio** | **p value** |
| Homing | After trap – Before trap | 0.020 | 0.004 | 818.081 | 5.234 | **< 0.001** |
| Foraging | After trap – Before trap | –0.017 | 0.004 | 819.590 | –4.582 | **< 0.001** |

**Table S7:** Post hoc comparisons of statistical results for scans across positions in the different directions (alpha = 0.01).

| **Scans** | | | | | | |
| --- | --- | --- | --- | --- | --- | --- |
| **Direction** | **contrast** | **estimate** | **SE** | **df** | **t ratio** | **p value** |
| Homing | After Trap – Before Trap | 0.797 | 0.264 | 781.105 | 3.016 | **0.011** |
| Foraging | After Trap – Before Trap | –4.447 | 0.247 | 781.105 | –17.967 | **< 0.001** |

**Table S8:** Post hoc comparisons of statistical results for scans across trips in the different directions (alpha = 0.01).

| **Scans** | | | | | | |
| --- | --- | --- | --- | --- | --- | --- |
| **Direction** | **contrast** | **estimate** | **SE** | **df** | **t ratio** | **p value** |
| Homing | Trip1 – Trip2 | –0.252 | 0.372 | 792.307 | –0.678 | 0.905 |
| Homing | Trip2 – Trip3 | –0.001 | 0.382 | 800.802 | –0.004 | 1.000 |
| Homing | Trip3 – Trip4 | 0.002 | 0.379 | 795.053 | 0.004 | 1.000 |
| Foraging | Trip1 – Trip2 | –2.047 | 0.350 | 782.138 | –5.846 | **< 0.001** |
| Foraging | Trip2 – Trip3 | –0.233 | 0.348 | 781.105 | –0.670 | 0.908 |
| Foraging | Trip3 – Trip4 | –1.389 | 0.350 | 782.138 | –3.966 | **< 0.001** |

**Table S9:** Post hoc comparisons of statistical results for scans across trips in the different positions (alpha = 0.01).

| **Position** | **contrast** | **estimate** | **SE** | **df** | **t ratio** | **p value** |
| --- | --- | --- | --- | --- | --- | --- |
| After Trap | Trip 1 – Trip 2 | –0.47 | 0.361 | 784.46 | –1.295 | 0.566 |
| After Trap | Trip 2 – Trip 3 | –0.214 | 0.365 | 786.849 | –0.587 | 0.936 |
| After Trap | Trip 3 – Trip 4 | –0.259 | 0.364 | 785.267 | –0.713 | 0.892 |
| Before Trap | Trip 1 – Trip 2 | –1.832 | 0.361 | 784.46 | –5.078 | **< 0.01** |
| Before Trap | Trip 2 – Trip 3 | –0.02 | 0.365 | 786.849 | –0.056 | 0.999 |
| Before Trap | Trip 3 – Trip 4 | –1.127 | 0.364 | 785.267 | –3.096 | **0.011** |
